# Nutrient composition of meals served to adult inpatients in public hospitals in North West, South Africa

**DOI:** 10.4102/hsag.v30i0.2842

**Published:** 2025-01-31

**Authors:** Mantombi J. Jiyana, Lindiwe J. Ncube

**Affiliations:** 1 Department of Mathematics, Sciences and Business Education, Faculty of Humanities, Tshwane University of Technology, Tshwane, South Africa; 2 School of Hospitality and Tourism Management, Faculty of Economics, Development and Business Sciences, University of Mpumalanga, Mbombela, South Africa

**Keywords:** normal diet meals, nutritionally adequate, meal quality, hospital-meals, nutrient-content

## Abstract

**Background:**

A high-quality meal is essential to patients’ food enjoyment and nutritional value. Patients’ health and nutritional status depend on the quality of meals provided by the hospital food service providers. Equally, patients are largely inactive and could experience energy imbalances if the ‘energy in’ exceeds the ‘energy out’, increasing the chances of the inception of obesity and obesity-related illnesses.

**Aim:**

This study assessed the nutrient content of meals served in selected public hospitals in the North West province, South Africa.

**Setting:**

The normal diet meals of four district hospitals and one regional hospital in the North West province were collected for this study.

**Methods:**

Regular adult meals for breakfast, lunch and supper including all snacks for the day were collected from five hospitals. Each edible food item was weighed and recorded on the weighed food records for later nutrient analysis using the South African-based Food Finder 3 software.

**Results:**

Meals in one hospital met Recommended Dietary Intake (RDI) (6829KJ) requirements for total energy, while in three hospitals, the requirements were exceeded, and in one hospital, meals were below RDI (5734KJ) requirements.

**Conclusion:**

Strict adherence to nutritionally adequate meals for hospitalised patients could positively influence and encourage patients to sustain healthy eating habits after discharge to prevent malnutrition.

**Contribution:**

This study provides relevant information that hospital managers can use to improve processes and procedures for dietary planning and ensure that hospital meals are nutritionally adequate.

## Introduction

Optimum nutrition is a basis for the preventative primary health care approach and one of the key developmental priorities in South Africa. In South African public hospitals, the food services department provides meals for patients. Hence, hospital meals contribute positively to patient’s health outcomes, quality of life and food enjoyment (Kraef et al. 2020). The World Health Organization (WHO 2014) suggests that good nutrition is consuming adequate, well-balanced meals comprising all essential nutrients required to sustain life (Espinosa-Salas & Gonzalez-Arias 2023; Sekumade 2013). Nutrients derived from meals are considered original medicine and the basis of modern medicine, subsequently they are prioritised instead of nutritional supplements. Therefore, it is important to ensure that all meals, including normal diets provided to inpatients, are nutritionally adequate. Historically, ill health is attributed to a poor and nutritionally inadequate diet, deficient in micro-nutrients and energy foods (Crino et al. 2015). Thus, optimal nutrition is critical for speeding up patient recovery, reducing malnutrition and related risks, and helping patients fight infection. Meeting the Recommended Dietary Intakes (RDIs) and serving nutritionally adequate meals and snacks to patients are important to improve clinical outcomes, reduce complications and rehospitalisation rates, and shorten hospital stays, ultimately saving elevated hospitalisation costs.

A normal diet meal is fundamental for hospitalised patients as all therapeutic diets are based and adapted from it; hence, most patients rely exclusively on hospital food for their nutritional requirements. Furthermore, it is imperative to ensure that meals served to hospitalised patients meet the RDIs. Different studies have been conducted in developed and developing countries to assess the nutritional adequacy of hospital menus and meals and reported mixed results. A study by Van Zwienen-Pot, Visser and Kruizenga (2018) in the Netherlands found that only a few older patients admitted to a nursing home have adequate protein and energy intake. Similarly, a study conducted in hospitals in Spain showed that energy, macronutrients, vitamins and minerals for diabetic and soft diet meals had insufficient vitamin E, D and magnesium as well as calcium, potassium, zinc and copper in some menus (Barcina-Pérez et al. 2023). Also, a study done in Switzerland’s hospitals showed that meals served to patients were below 1500 kcal/day, and lacked sufficient iron, zinc, thiamine, vitamin B12 and vitamin C (Berger et al. 2019). In addition, a study conducted by Trang et al. (2015) in hospitals in Canada revealed that 45% of the menus were lower than the Canadian Dietary reference intake (DRI) for total energy, while protein was higher than the DRI requirements. Contrarily, a study conducted in hospitals in Greece by Tsagari (2016) found that meals offered at hospitals met the energy, protein and food group servings, except dairy products according to the European guidelines. Equally, a study conducted in a hospital in Turkey evaluating the micronutrient status of hospitalised patients in an infectious disease clinic found that patients were deficient in zinc, selenium, thiamine, vitamin B6 and vitamin B12, and most of them had multiple micronutrient deficiencies (Dizdar et al. 2016). In addition, a study by Boutata et al. (2022) in Algeria found that energy intakes were deficient, and protein intakes were lower than the dietary reference values (DRV).

South African public hospitals use a ration scale developed by the South African Department of Health to ensure hospitalised patients receive meals providing sufficient energy and macro- and micro-nutrients. The ration scales are based on the South African Food-Based Dietary Guidelines (FBDG) adopted from the WHO dietary guidelines, and the RDIs, including the five food groups (Nestle 2024; Vorster, Badham & Venter 2013). A study conducted by Jiyana et al. (2016) in hospitals in Gauteng province, South Africa, showed that only two out of the eight hospitals’ adult normal diet menus met the RDI; four were below the RDIs, and two hospitals’ menus exceeded the RDIs. In addition, a study conducted at a tertiary hospital outpatient clinic in Tshwane District, South Africa, reported that patients with type 2 diabetes mellitus had insufficient energy intake, and intakes for vitamin D, calcium, folate and iron were below half of the RDI. Furthermore, a study conducted in three public hospitals in Cape Town metropole found that the total energy and protein of meals served to patients were below the RDIs (Theron & O’Halloran 2021).

Drawing from the above-discussed studies, only a few hospital meals meet the DRIs. Although the studies done in different countries were not based only on normal diets, certainly, most meals served to hospitalised patients do not meet the RDIs. Efficient menu planning guarantees the provision of nutritionally adequate meals. However, if inadequate food portions are served, patients’ nutritional needs will not be met. Hence, the vision of a policy developed by the South African National Department of Health (SADoH 2015) is to provide optimal nutrition for all patients in public health establishments in South Africa. This vision tasks the food service managers to procure, prepare and serve nutritionally adequate meals to diversified hospital patients to reduce the risks of chronic diseases and maximise their recovery levels (Brown & Phillips 2010). Globally, including in South Africa, studies on the nutritional adequacy of normal diet meals served to hospitalised patients are scarce. The nutritional quality of normal diet meals served in most hospitals in South Africa is unknown. This study assessed the nutrient content of meals served to inpatients in public hospitals in North West province, South Africa.

## Research methods and design

### Study design

This study applied a cross-sectional quantitative, descriptive, analytical approach to assess the nutrient composition of normal diet meals served to adult inpatients in selected public hospitals in North West province, South Africa

### Study population and sampling strategy

North West has 19 district and 4 regional hospitals. A stratified random sampling strategy was used to select four district and four regional hospitals for the study. Four district hospitals and one regional hospital were permitted to conduct the study. Each hospital’s 1-day meal consisting of food items for breakfast, lunch, supper and all the snacks provided to inpatients were weighed separately and recorded for later analysis. Pre-plated normal diet meals for medical and general wards were randomly selected in a food service facility dishing-up area.

### Data collection

Data were collected from one regional and four district hospitals that used outsourced catering services. Edible normal diet items for breakfast, lunch, supper and adult male and female patients’ snacks for a day, were randomly selected from the dishing-up area, weighed, and weights recorded on the data record sheet for subsequent analysis. An ADAM LBK30 electronic scale was used to weigh food portion sizes to be served for breakfast, lunch, supper and snacks on the day of the visit. The scale was calibrated, the plate used for serving food was weighed and the weight was subtracted from each weight of the food item weighed to ensure accuracy. The researcher weighed three samples of each food items served by the hospital for breakfast, lunch, supper, snacks and drinks. All individual food portions (e.g., chicken, rice, and vegetables) were weighed by plating food items onto a plate and recording the weight of each food item separately using an electronic digital scale. The average amount of each food item plated on three plates was used to calculate the amount served per mealtime.

### Reliability

An Adam LBK30 electronic scale was used to weigh food items, and each time the food items were weighed, the scale was calibrated to zero to ensure the reliability of the data collected and analysed. Each food item was weighed three times and the average weight was calculated to ensure accuracy.

### Validity

A South African-based Food Finder3 software program developed by the Medical Research Council (MRC), validated and widely used in previous studies (MRC 2015) to analyse nutrients, was used in this study to assess the nutrient content of the hospital meals.

### Data analysis

Data were analysed by the researcher using Food Finder3, which is the latest version of the South African Food Composition Database (SAFOODS) software. The nutrient content was assessed based on the current DRIs for macro and micro nutrients. Mean values and standard deviations were analysed for the total energy, protein, carbohydrate, fat, iron, vitamin A, folate, vitamin B_6_ and vitamin B12.

### Ethical considerations

Ethical approval was obtained from the Tshwane University of Technology Ethics Committee (Ethical clearance no: Ref# 2009/08/003). The National Department of Health, the Northwest Health Department, and the chief executive officers (CEOs) of the public hospitals that participated in the study granted approval and permission for the study.

## Results

The nutrient composition of normal diet meals served in five public hospitals in North West was analysed for total energy, protein, fat, carbohydrate, iron, folate, vitamin B_6_ and content, and measured against the South African DRI standards.

### The total energy composition of normal diet meals served in five public hospitals in North West province

**FIGURE 1:**
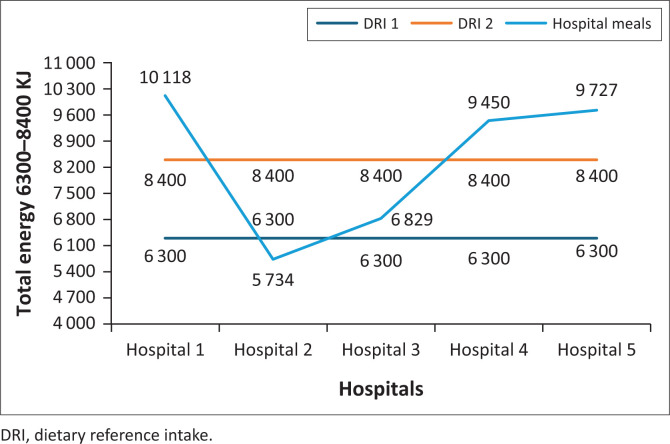
Total energy content of normal diet meals served in public hospitals in North West province.

The RDI for total energy is 6300 kJ – 8400 kJ. Of the normal diet meals served to adult patients in five public hospitals, only one hospital meal met the RDI requirements, three were above and one was below the total energy RDI (Figure 1).

### The protein content of normal diet meals served in five public hospitals in North West province

**FIGURE 2:**
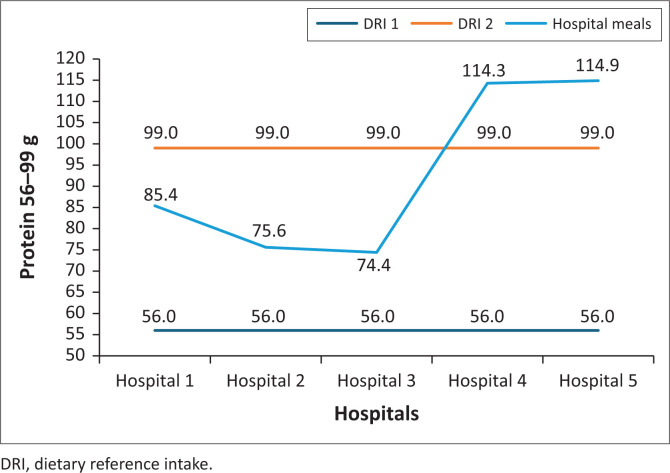
Protein content of normal diet meals served in five public hospitals in North West province.

Meals served to patients met the RDIs for protein (56 g – 99 g) in three hospitals and were above the RDIs in two hospitals (Figure 2).

### The carbohydrate content of normal diet meals served in five public hospitals in North West province

**FIGURE 3:**
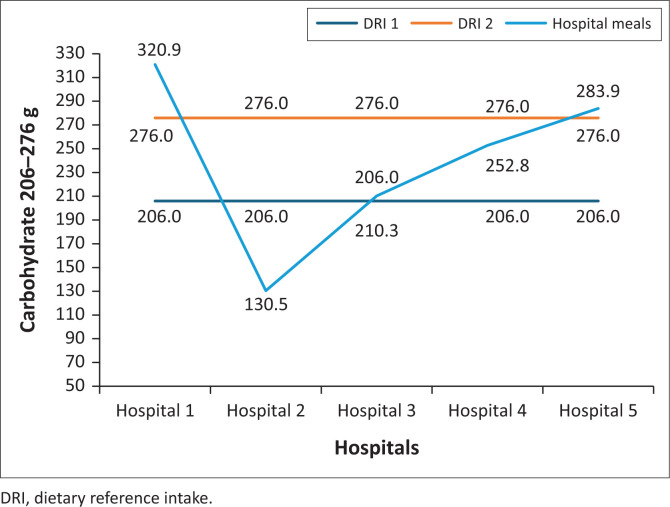
Carbohydrate content of normal diet meals served in public hospitals in North West province.

Normal diet meals in two hospitals met the RDI (206 g – 276 g) for carbohydrates, two hospitals’ meals exceeded the RDI, and one hospital’s meal was below the RDI (Figure 3).

### The fat content of normal diet meals served in five public hospitals in North West province

**FIGURE 4:**
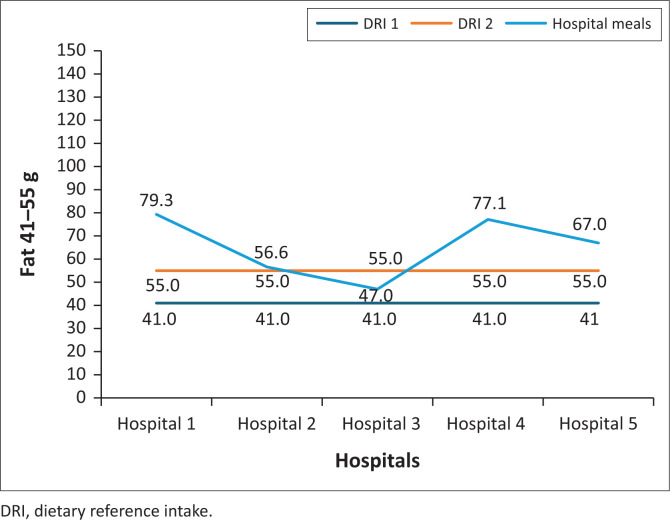
Fat content of normal diet meals served in five public hospitals in North West province.

Normal diet meals in four hospitals were above the RDI (41 g – 55 g) for fat, and one hospital met the RDI (Figure 4).

### The iron content of normal diet meals served in five public hospitals in North West province

**FIGURE 5:**
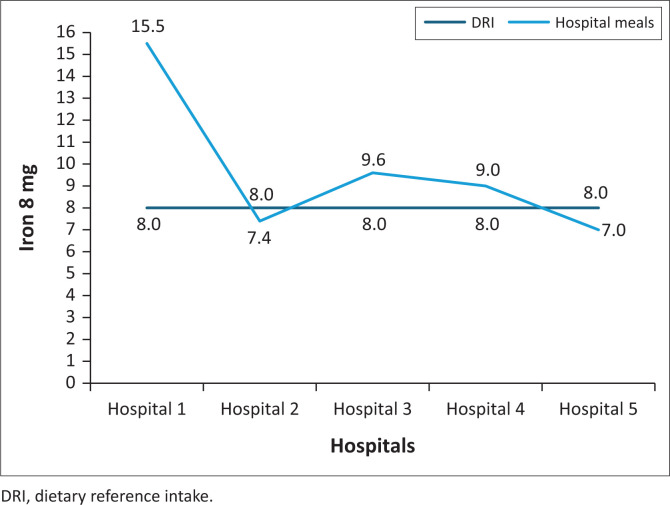
Iron content of normal diet meals served in five public hospitals in North West province.

None of the hospital’s meals met the 8 mg RDI for iron, with two below and three above the RDI (Figure 5).

### The folate content of normal diet meals served in five public hospitals in North West province

**FIGURE 6:**
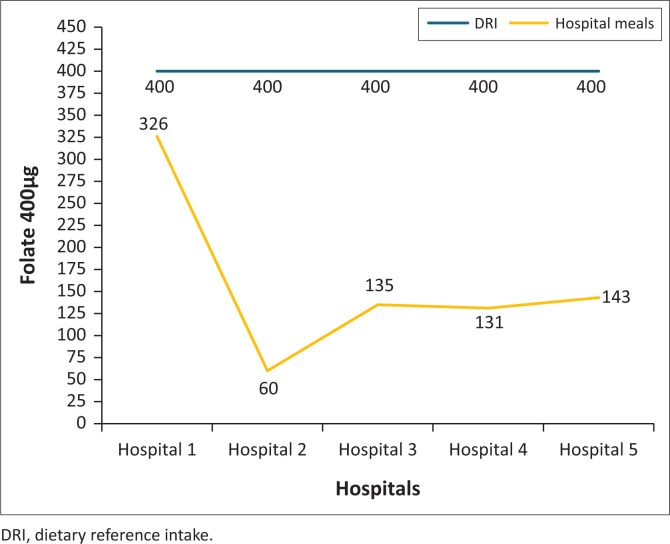
Folate content of normal diet meals served in five public hospitals in North West province.

None of the hospital meals met the RDI of 400 µg for folate and all the hospitals’ meals were below the RDI (Figure 6).

### The vitamin A content of normal diet meals served in five public hospitals in North West province

**FIGURE 7:**
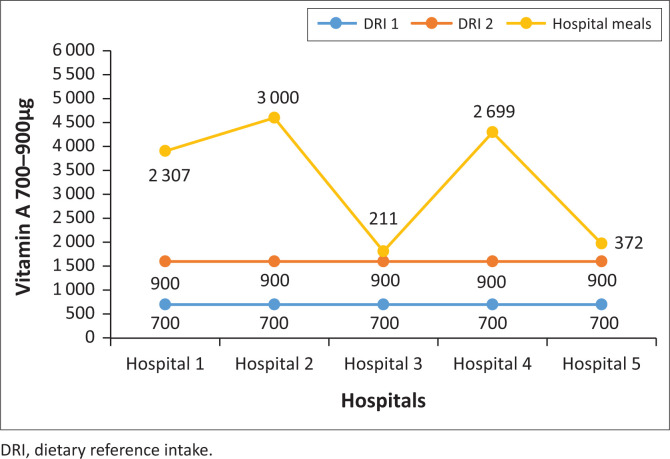
Vitamin A content of normal diet meals served in five public hospitals in North West province.

Two hospitals’ normal diet meals were below the RDI of 700 µg – 900 µg for vitamin A, and those from three hospitals were above RDI. None of the hospitals met the RDI for vitamin A (Figure 7).

### The vitamin B_6_ content of normal diet meals served in five public hospitals in North West province

**FIGURE 8:**
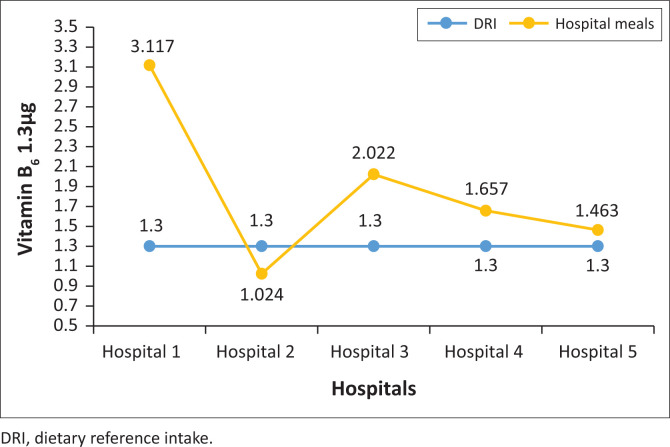
Vitamin B_6_ content of normal diet meals served in public hospitals in North West province.

Normal diet meals in one hospital were below the RDI of 1.3 µg for vitamin B_6_, and meals in four hospitals were above the RDI (Figure 8).

## Discussion

### Macronutrient content of meals served to patients in public hospitals in the North West province

This study assessed the nutrient composition of normal diet meals in public hospitals in the North West province using the SAFOODS software (Food Finder 3). Macronutrients (fats, proteins and carbohydrates) are needed in larger quantities and provide the human body with energy.

For most hospitals in this study, there were inconsistencies in the macronutrient content of normal diet meals served to hospitalised patients. The meal’s total energy content exceeded the RDIs. Only one hospital meal met the RDI and one was below the RDI; two hospitals’ meals were above the RDI for carbohydrates and one was lower.

Most hospitals’ meals in this study met the protein RDI, but only two hospitals served meals with protein content higher than the RDI. Similarly, Trang et al. (2015) found that menus planned for Canadian hospitals were lower than the DRI for total energy and protein intakes were higher than the DRI. Also, Van Zwienen-Pot et al. (2018) found that few older adults admitted to a nursing home in the Netherlands had adequate energy and protein intake. In addition, a study conducted in Switzerland showed that hospital meals provided to patients were deficient in total energy (Berger et al. 2019). Contrarily, a study conducted by Barcina Perez et al. (2023) in Spain reported that hospital meals met the total energy RDIs; and Tsagari (2016) in Greece found that meals served in hospitals met both the total energy and protein RDIs. In Jordanian hospitals, the total energy content of the normal diet meal was less than 2000 Kcal (El-Qudah 2018).

Studies conducted in the African continent showed similar results. For example, meals were lower in total energy and protein DRV in hospitals in Algeria (Boutata et al., 2022). In addition, in a study conducted in three public hospitals in Cape Town metropole, South Africa, the total energy and protein of normal diet meals served to patients were below the RDIs (Theron & Halloran 2021). In addition, a study conducted by Jiyana, Ncube and Nesamvuni (2018) in public hospitals in Gauteng province, showed that normal diet menus were deficient in total energy. Furthermore, a study done at a tertiary hospital outpatient clinic in Tshwane district, South Africa, reported that patients with type 2 diabetes mellitus had insufficient energy intake. Hence, the FBDG recommend that dried beans, grains and pulses be included in daily meals to increase fibre intake for meals deficient in carbohydrates (Vorster et al. 2013). Proteins are important for hospitalised patients consuming normal diet meals because they reduce infectious complications and spontaneous wounds and prevent muscle loss thus reducing the length of hospital stay (Deutz et al. 2014; Dijxhoorn et al. 2021).

Insufficient food intake in hospitals is an important factor in the development of malnutrition. Most patients admitted are already malnourished and can be further perpetuated in a hospital because of the provision of nutritionally inadequate meals. The imbalances of the total energy and deficient RDI could be attributed to poorly planned menus, deviation from properly planned menus and poor portion control (Viganó et al. 2011). Sufficient nutrient provision to hospitalised patients will be assured if the food portion sizes are accurately controlled.

Four hospital meals in this study were above the fat RDI and one was below. Defiantly, Ncube (2012) observed a low-fat content in meals served to patients in 13 South African public hospitals. Inadequacies in the fat content indicate that hospitalised patients are offered meals high in saturated fatty acids and trans-fatty acids, which could increase low-density lipoprotein (LDL) cholesterol levels, and, in turn, increase the risk of heart disease. There is an ongoing debate regarding whether replacing saturated fat with unsaturated fat in meals yields health benefits to patients. The American Heart Association Presidential Advisory recommends lowering saturated fat intake and replacing it with polyunsaturated fats to decrease cardiovascular disease (CVD) (Sacks, Lichtenstein & Wu 2017). Contrarily, Dehghan et al. (2017) found that higher total saturated fat intakes (monounsaturated and polyunsaturated fatty acids) were not associated with CVD mortality or incidence, except for inverse associations of saturated fatty acids with the incidence of stroke but with lower total mortality.

### Micronutrient content of meals served to patients in public hospitals in the North West province

This study showed that meals in two hospitals were below the RDI for iron and three were above the RDI for iron; all the hospitals provided meals with lower folate content; none of the hospital meals met the vitamin A RDI. Meals at two hospitals were below the vitamin A RDI and at three hospitals were above the vitamin A RDI and high vitamin B_6_ content meals were served to patients. None of the hospital meals met the vitamin B_6_ RDI. Studies showed insufficient folate in meals served to hospitalised patients (Slavin & Lloyd 2012; Storey & Anderson 2014; Tiwari & Cummins 2013). Similarly, a study conducted in eight public hospitals in Gauteng province showed that the hospitals ‘planned menus did not meet the iron and folate RDIs. Six hospitals were below and two were above the vitamin A RDI; five hospitals’ menus were below vitamin B_6_ and three were above. In addition, El-Qudah (2018) found that all hospitals provided meals low in vitamin A and did not meet the nutritional standards. In addition, a study conducted at a tertiary hospital outpatient clinic in Tshwane district, South Africa, reported that the folate and iron intakes of patients with type 2 diabetes mellitus were below half the RDI.

Iron is a micronutrient necessary for the body’s growth and development. The human body uses iron to make haemoglobin – a protein in red blood cells that carries oxygen from the lungs to the body and myoglobin – which provides oxygen to muscles. Therefore, inadequate iron content in the served meals results in the iron stored in the body decreasing and the cells failing to carry sufficient oxygen from the lungs to the body tissues resulting in anaemia, impaired immunity, and unregulated body temperature (Rolfes, Pinna & Whitney 2015). Consequently, patients consuming meals with insufficient iron are at increased risk of iron deficiency.

Folate is a micronutrient mostly found in dark green, leafy vegetables, legumes and fruits, especially citrus fruits and juices. Fruits and vegetables have anti-carcinogenic effects and phytochemicals that decrease the oxidative damage that accounts for the onset of most chronic diseases. Some of the effects associated with inadequate folate intake are a high risk of birth defects, increased pancreatic cancer risk, anaemia and heart disease (Rolfes et al. 2015). Therefore, adequate consumption of vegetables and citrus fruits may prevent chronic diseases.

Vitamin A is a micronutrient found in fruits, vegetables and fish. Carotenoids are a group of chemicals found in plants; they are needed for the proper growth and functioning of body parts including eyes and skin, and boosts the immune system. Consumption of a vitamin A-deficient diet causes infection because of severely weakened immunity that could lead to death (Wiseman, Bar-El Dadon & Reifen 2017). Respiratory and gastrointestinal stability is compromised as immunity weakens, possibly leading to chronic diarrhoea (Correia et al. 2016). Prins (2010) alluded that meals deficient in vitamin A, iron and other macronutrients lead to severe infections linked to decreased wound healing and postoperative complications.

Vitamin B_6_ (pyridoxine) is important for normal brain development and keeping the nervous and immune system healthy. Food sources of vitamin B_6_ include poultry, fish, potatoes, chickpeas, bananas and fortified cereals. Equally, as people age their vitamin B_6_ levels decline. Poor vitamin B_6_ intake is linked to an increased risk of heart disease, rheumatoid arthritis, Alzheimer’s disease and other forms of dementia (Wu et al. 2008). In addition, an increase in cases of cancer of the trachea, bronchi, lung, oesophageal and prostate in males including an increase in cancer of the cervix, breast and lung in females, is attributed to lower vitamin B_6_ levels (Naude 2013). Failure to consume sufficient vitamin B_6_ may lead to nerve damage that manifests in numbness and muscle weakness and ultimately, inpatients may end up suffering from depression. An irritable, depressed patient is likely to have a decreased appetite, which can lead to malnutrition and a longer hospital stay (Rolfes et al. 2015).

### Strengths and limitations

One of the study’s strengths is using the SAFOODS software (Food Finder 3) which ensured the validity of the questionnaire. The study was conducted in selected public hospitals in North West province and can be replicated in other public hospitals in the country.

### Implication of the study

This study provides valuable insights for hospital managers aiming to enhance dietary planning processes and procedures, ensuring the nutritional adequacy of hospital meals. Concurrently, it emphasises the need to reassess hospital-catering budgets to avoid compromising patients’ dietary requirements because of financial constraints. Based on these findings, administrators must allocate sufficient resources to ensure menu planning, procurement, preparation, holding, portioning and serving processes to maintain optimal nutrient content. Rigorous controls should be implemented to ensure meals are portioned according to RDIs, providing patients with essential nutrients crucial for health and recovery from illness.

Hospital management teams must prioritise food services alongside medication, recognising their synergistic role in patient care. Effective collaboration between food service managers and dieticians is essential to communicate and meet patients’ dietary needs based on their conditions, age, gender and national dietary guidelines.

## Conclusion

The findings of this study enhance understanding of the crucial role that nutritionally adequate meals play for hospitalised patients. They highlight the necessity of providing appropriate resources to ensure that menu planning, procurement, preparation, holding, portioning and serving processes maintain nutrient integrity. Hospital managers can leverage these findings to identify gaps and inconsistencies in meal provision, as well as to establish and implement food service policies that guide everyone involved in the hospital food chain –from procurement and storage to production, serving, distribution and delivery. This will help ensure that patients receive meals in sufficient quantities and with the necessary nutrients to prevent malnutrition during their hospital stay.

In addition, these findings can inform improvements in dietary planning and budgeting processes, as catering budgets significantly influence hospital food provision. It is essential that patients’ dietary needs are prioritised and not compromised because of financial constraints.
